# EP Fellows Summit 2025: Case Competition Finalists

**DOI:** 10.19102/icrm.2025.17047

**Published:** 2026-04-15

**Authors:** 

**Keywords:** Atrial fibrillation, left ventricular assist device, pulsed field ablation, ventricular arrhythmia

## Abstract

The 2025 EP Fellows Summit & Arrhythmia Scholars Program was held November 7–9, 2025, in Reston, Virginia.

## Abstract 1

DOI: 10.19102/icrm.2025.170471

### A Case Report of Persistent Right Phrenic Nerve Palsy Following Pulsed Field Ablation in a Patient with Persistent Atrial Fibrillation

Hasan Munshi, MD,^1^ Abdullah Ahmad, MD, MPH,^1^ Abdel Dajani, MD,^1^ Own Khrasiat, MD,^2^ Amer Hammad, MD,^2^ and Satish Tiyyagura, MD, FACC, FHRS^1^

^1^St. Joseph’s University Medical Center, Paterson, NJ, USA

^2^Englewood Hospital and Medical Center, Englewood, NJ, USA

The authors report no conflicts of interest for the published content. No funding information was provided.

Corresponding author: Abdullah Ahmad, MD, MPH; abdullah.s.ahmad@hotmail.com

***Introduction:*** Pulsed field ablation (PFA) is a nonthermal modality that achieves myocardial-specific ablation via irreversible electroporation while limiting injury to adjacent structures. Large clinical trials have shown noninferiority to thermal ablation, with fewer adverse events. Phrenic nerve (PN) injury, although rare, remains a risk and may be influenced by catheter location relative to the PN. We present a case of persistent right PN palsy after pulmonary vein isolation (PVI) and posterior wall isolation (PWI) using a PulseSelect™ (Medtronic, Inc., Minneapolis, MN, USA) catheter in a patient with persistent atrial fibrillation (AF).

***Case presentation:*** A 61-year-old woman with persistent AF (diagnosed in 2020), hypertension, hypothyroidism, and a prior coronavirus disease 2019 infection had recurrent symptomatic AF despite anti-arrhythmic therapy. She declined amiodarone because of side-effect concerns. Due to dizziness, 100 mg of flecainide twice daily was reduced to 50 mg twice daily. Anticoagulation was changed from apixaban to dabigatran for affordability.

Pre-procedural testing showed a normal echocardiogram, a baseline electrocardiogram (ECG) demonstrated AF, and the chest radiograph was normal **(Figure 1)**. She underwent catheter ablation using PFA.

**Figure 1: fg001:**
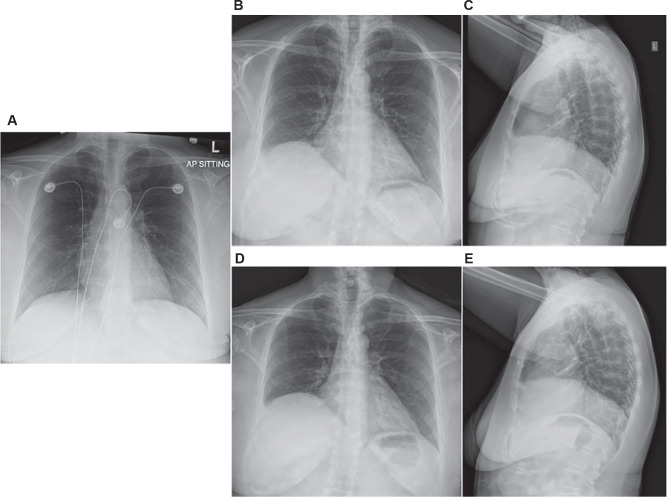
Chest X-ray images. **A:** Normal antero-posterior chest radiograph pre-ablation. **B:** Post-procedural antero-posterior chest radiograph showing right hemidiaphragm elevation consistent with diaphragm paralysis. **C:** Post-procedural lateral chest radiograph showing right hemidiaphragm elevation consistent with diaphragm paralysis. **D:** Post-procedural 3-month follow-up antero-posterior chest radiograph showing right hemidiaphragm elevation consistent with diaphragm paralysis. **E:** Post-procedural 3-month follow-up lateral chest radiograph showing right hemidiaphragm elevation consistent with diaphragm paralysis.

Under general anesthesia, ultrasound-guided venous access was obtained. A SoundStar® (Biosense Webster, Diamond Bar, CA, USA) intracardiac echocardiography catheter showed no thrombus or effusion. A single transseptal puncture was performed with a FlexCath sheath (Medtronic, Inc.). Left atrial voltage mapping was completed using an Octaray™ catheter (Biosense Webster) cathter on CARTO™ 3 (Biosense Webster) **(Figure 2)**. PFA with a Medtronic PulseSelect™ catheter achieved circumferential PVI of all four veins. Because of fractionated electrograms on the posterior wall, linear lesions were applied across the superior and inferior pulmonary veins to complete PWI. Entrance and exit blocks were confirmed for all pulmonary veins and the posterior wall. No pericardial effusion occurred. The total fluoroscopy time was 3 min.

**Figure 2: fg002:**
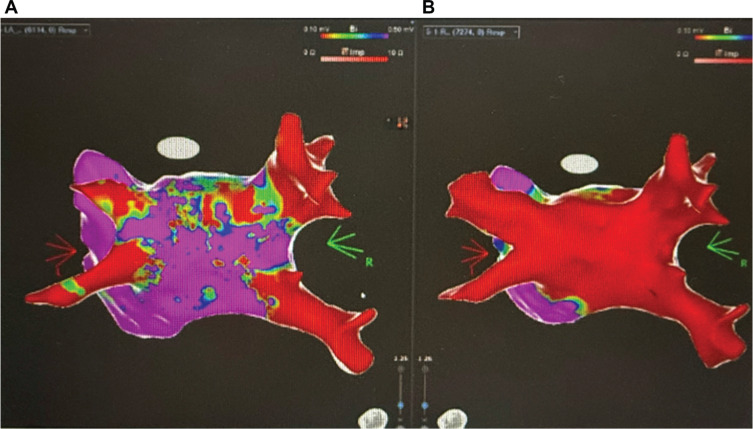
Voltage mapping. **A:** Pre-ablation voltage mapping showing heterogeneous atrial substrate with preserved and low-voltage areas. **B:** Post-ablation voltage mapping demonstrates extensive low-voltage areas consistent with successful lesion ablation.

The patient was discharged the same day. Pre-procedure dizziness resolved, but new mild shortness of breath began a few hours after ablation and persisted. Chest radiographs obtained several days later showed elevation of the right hemidiaphragm, consistent with right PN palsy **(Figures 1A–1C)**. Conservative management with incentive spirometry was initiated. At 12-day follow-up, dyspnea was improving. ECG showed normal sinus rhythm with occasional premature atrial contractions; 50 mg of flecainide twice daily and dabigatran were continued. At 3 months, chest radiography demonstrated persistent elevation of the right hemidiaphragm **(Figures 1D and 1E)**, confirming ongoing PN palsy with mild functional symptoms.

***Discussion:*** This appears to be among the first reports of persistent PN paralysis associated with the PulseSelect™ PFA system. Although PFA aims to spare non-cardiac tissues, PN injury can occur when energy is delivered near the right pulmonary veins, where the PN runs in close proximity. In the MANIFEST-PF (Multi-National Survey on the Methods, Efficacy, and Safety on the Post-Approval Clinical Use of Pulsed Field Ablation) registry of 1568 patients treated with the FARAPULSE system (Boston Scientific, Marlborough, MA, USA), transient PN injury occurred in 0.4% (typically resolving within a day),^1^ and permanent paralysis was documented in 0.06%.^2^ The PULSED AF (Pulsed Field Ablation to Irreversibly Electroporate Tissue and Treat Atrial Fibrillation) pivotal trial of PulseSelect™ (N = 300) demonstrated durable PVI without persistent PN palsy.^3^ Early experience with VARIPULSE (Biosense Webster), integrated with CARTO™ 3, similarly showed high acute success without persistent PN involvement.^4^ The ElectroPulse study of a novel 10-electrode variable-loop PFA catheter performing PVI plus PWI in 30 patients reported no PN palsy^5^; however, the sample size was small, and longer follow-up is needed. Collectively, PFA is largely tissue-selective, yet vigilance for PN injury remains important.

Mechanisms of PN injury after PFA are incompletely understood. Animal studies have described small hemorrhages and perineural edema with preserved nerve architecture, supporting a nonthermal, electrophysiologic effect rather than frank thermal necrosis. Compound motor action potential (CMAP) monitoring may detect intraprocedural PN dysfunction, but validated amplitude thresholds are lacking, and CMAP is impractical when paralytic agents are used. In a cohort using an off-label pentaspline PFA catheter for pulmonary vein and superior vena cava isolation, transient PN “stunning” detected by CMAP occurred in 64% of patients without post-procedure palsy on imaging, supporting a reversible electrophysiologic effect in most cases.^6^ Our patient’s persistent deficit emphasizes that durable dysfunction, while uncommon, can occur.

***Conclusion:*** Even with the tissue-selective nature of PFA, persistent right PN palsy can occur during PVI plus PWI. Peri-procedural vigilance, attention to anatomic proximity, conservative management, and structured follow-up are essential to optimize outcomes.

## References

Ekanem E, Reddy VY, Schmidt B, et al. Multi-national survey on the methods, efficacy, and safety on the post-approval clinical use of pulsed field ablation (MANIFEST-PF). *Europace.* 2022;24(8):1256–1266. [CrossRef]
[PubMed]Ekanem E, Neuzil P, Reichlin T, et al. Safety of pulsed field ablation in more than 17,000 patients with atrial fibrillation in the MANIFEST-17K study. *Nat Med.* 2024;30(7):2020–2029. [CrossRef]
[PubMed]Verma A, Haines DE, Boersma LV, et al. Pulsed field ablation for the treatment of atrial fibrillation: PULSED AF pivotal trial. *Circulation.* 2023;147(19):1422–1432. [CrossRef]
[PubMed]Duytschaever M, De Potter T, Grimaldi M, et al. Paroxysmal atrial fibrillation ablation using a novel variable-loop biphasic pulsed field ablation catheter integrated with a 3-dimensional mapping system: 1-year outcomes of the multicenter inspIRE study. *Circ Arrhythm Electrophysiol.* 2023;16(3):e011780. [CrossRef]
[PubMed]Kamsani SH, Emami M, Young GD, et al. First-in-human experience of high-energy ElectroPulse pulsed field ablation: acute results for pulmonary veins and posterior wall isolation. *Heart Rhythm.* 2025;22(8):e309–e317. [CrossRef]
[PubMed]Ollitrault P, Chaumont C, Font J, et al. Superior vena cava isolation using a pentaspline pulsed-field ablation catheter: feasibility and safety in patients undergoing atrial fibrillation catheter ablation. *Europace.* 2024;26(7):euae160. [CrossRef]
[PubMed]

## Abstract 2

DOI: 10.19102/icrm.2025.170472

### Purkinje Fiber Catheter Ablation for Incessant Ventricular Fibrillation in a Patient with a Left Ventricular Assist Device

Jonathan S. Gordon, MD,^1^ Raktham Mekritthikrai, MD,^1^ and Henry D. Huang, MD^1^

^1^Rush University Medical Center, Chicago, IL, USA

The author reports no conflicts of interest for the published content. No funding information was provided.

Corresponding author: Jonathan Gordon, MD; jongordon275@gmail.com

***Background:*** Abnormal Purkinje fiber potentials and activity have been implicated in the initiation of ventricular fibrillation (VF) in both myocardial ischemia and idiopathic VF. Their role in the generation of VF and Purkinje-mediated fast ventricular tachycardias (VTs) in those with structural heart disease is not clearly defined.

***Case presentation:*** A 73-year-old man with a history of nonischemic cardiomyopathy with a left ventricular (LV) assist device presented with appropriate implantable cardioverter-defibrillator therapies for recurrent episodes of fast VT and VF. A review of the presenting electrogram showed fast VT/VF initiated with a short-coupled (290 ms) premature ventricular complex (PVC) **(Figure 1A)**. He failed medical therapy with amiodarone, which was discontinued due to prolonged QT interval, and a maximally tolerated β-blocker.

**Figure 1: fg003:**
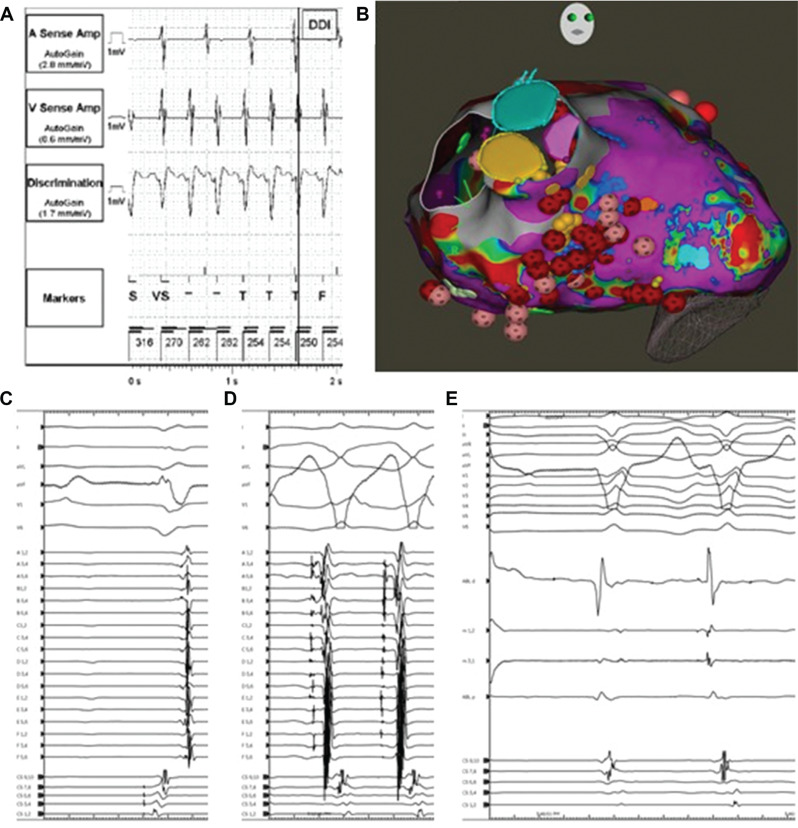
**A:** Presenting electrogram from the device showing the initiation of fast ventricular tachycardia/ventricular fibrillation with a short-coupled (<300 ms) premature ventricular complex. **B:** The CARTO™ 3 voltage map with high-power, short-duration ablation lesions at sites of abnormal Purkinje activity. **C:** Electrogram from the Optrell™ catheter showing low-voltage, fractionated Purkinje potentials. **D:** Fast ventricular tachycardia with pre-systolic Purkinje activity initiated with mechanical contact to areas with abnormal Purkinje potentials. **E:** Purkinje firing during ablation of these sites.

An attempted VT ablation was performed under general anesthesia using the CARTO™ 3 (Biosense Webster, Diamond Bar, CA, USA) mapping system and QDOT radiofrequency (RF) ablation catheter (Biosense Webster). Initially, ablation of the proximal portion of the right bundle branch was performed. Voltage mapping of the LV using an Optrell™ catheter (Abbott, Chicago, IL, USA) did not reveal a large area of scar **(Figure 1B)**. Purkinje potentials were present in the LV septum in the expected distribution of the anterior and posterior fascicles **(Figure 1C)**. Mechanical contact in these areas resulted in fast VT with pre-systolic Purkinje potentials **(Figure 1D)**. These areas were ablated with high-power, short-duration RF applications at a target of 90 W for 4 s. Application of ablation lesions terminated the runs of VT with pre-systolic Purkinje potentials caused by mechanical contact **(Figure 1E)**. Afterward, VT and VF were not inducible despite aggressive extra-stimuli with S2s delivered on T-waves and burst pacing down to 180 ms. The patient has remained free of VT/VF since ablation.

***Discussion:*** Short-coupled PVCs (<300 ms) originate from the Purkinje fibers and were this patient’s VF trigger. High-power, short-duration RF ablation is a novel approach that can debulk the Purkinje fibers by rapidly creating large, shallow lesions. This anatomical approach to ablation may reduce procedural time, especially important in those at high risk for decompensation and those on mechanical support.

***Conclusion:*** Catheter ablation of Purkinje fibers is a novel and promising approach to the treatment of VF, even in those with advanced cardiomyopathy with structural heart disease.

## Abstract 3

DOI: 10.19102/icrm.2025.170473

### Feasibility of Combined Pulsed Field and Radiofrequency Ventricular Tachycardia Ablation Using Sphere-9™ Guided by EnSite™ Mapping in a Patient with Durable Left Ventricular Assist Device

Sittinun Thangjui, MD^1^ and David Schwartzman, MD^1^

^1^Department of Cardiovascular Disease, Heart and Vascular Institute, West Virginia University, Morgantown, WV, USA

The authors report no conflicts of interest for the published content. No funding information was provided.

Corresponding author: Sittinun Thangjui, MD; s.thangjui@gmail.com

***Background:*** Ventricular tachycardia (VT) in patients with durable left ventricular assist devices (LVADs) presents significant procedural and mapping challenges. Electromagnetic interference (EMI) from the LVAD motor often precludes the use of magnetic-based mapping systems, complicating substrate identification and catheter localization.^1^ The Affera Sphere-9™ system (Medtronic, Minneapolis, MN, USA) is a novel ablation catheter capable of both radiofrequency (RF) and pulsed field ablation (PFA) energy delivery with integrated mapping functionality. However, the Affera platform’s electromagnetic field–based tracking becomes unreliable in patients with LVADs.^2^ We report the first successful case of VT ablation using Sphere-9™ for combined PFA and RF ablation guided by the EnSite™ impedance-based mapping system (Abbott, Chicago, IL, USA) in a patient with a HeartMate III™ LVAD (Abbott), demonstrating the feasibility and safety of this hybrid approach.

***Case presentation:*** A 70-year-old man with ischemic cardiomyopathy and end-stage heart failure with reduced ejection fraction (<20%) presented with acute-on-chronic decompensated heart failure and cardiogenic shock. His past medical history included coronary artery disease status post three-vessel coronary artery bypass grafting, multiple percutaneous coronary interventions to both native vessels and vein grafts, paroxysmal atrial fibrillation (on apixaban), obstructive sleep apnea (on continuous positive airway pressure), chronic kidney disease stage IV, and a dual-chamber implantable cardioverter-defibrillator (ICD). He was admitted for worsening symptoms and hemodynamic instability, requiring intra-aortic balloon pump placement on September 19, 2025, followed by durable LVAD (HeartMate III™) implantation.

The postoperative course was complicated by VT storm **(Figure 1)** requiring intravenous amiodarone and lidocaine infusions, right ventricular (RV) failure requiring temporary RV assist device (RVAD) support, and acute kidney injury requiring continuous renal replacement therapy (CRRT). Despite maximal medical therapy and recurrent ICD shocks, he continued to experience sustained VT episodes. Catheter ablation was therefore pursued.

**Figure 1: fg004:**
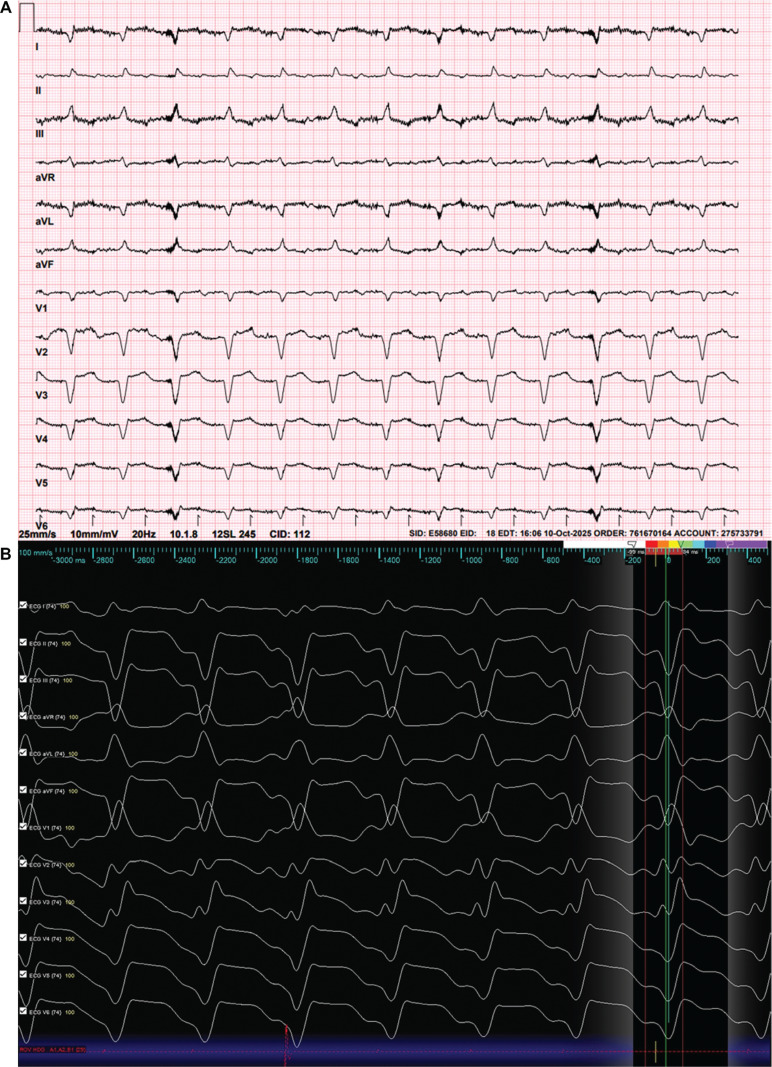
**A:** Baseline electrocardiogram. **B:** Ventricular tachycardia.

Bilateral femoral venous and left femoral arterial access were obtained under fluoroscopic and ultrasound guidance. The patient was systemically anticoagulated, and transesophageal echocardiography confirmed severe biventricular dysfunction without valvular pathology. The mechanical circulatory support team continuously monitored LVAD and RVAD performance.

Retrograde aortic access was obtained via the left femoral artery. Intracardiac echocardiography (ICE) guided transseptal puncture for left atrial and left ventricular (LV) access. Two catheters were used: an HD Grid catheter (Abbott) for detailed substrate mapping and localization and a Sphere-9™ catheter for lesion delivery **(Figure 2)**. Because of significant EMI, the Affera mapping system could not track the Sphere-9™ catheter, but impedance-based localization via the Abbott EnSite™ X system remained fully functional. Thus, the HD Grid–generated map was used to identify target sites, and the Sphere-9™ catheter was navigated under fluoroscopy and ICE guidance to deliver ablation at those mapped locations **(Figure 3, Video 1)**.

**Figure 2: fg005:**
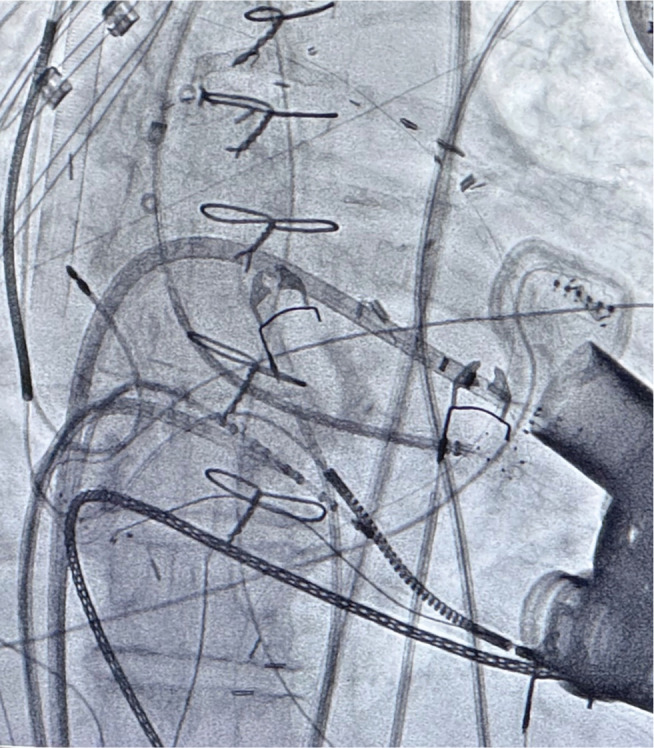
Fluoroscopic image of catheter positioning during the procedure. The HD Grid catheter was introduced via the Agilis (Abbott) sheath through transseptal access and the Sphere-9™ catheter was introduced through retrograde transaortic access. Dual-chamber implantable cardioverter-defibrillator, CentriMag (Abbott) right ventricular assist device, and durable HeartMate III left ventricular assist device were noted.

**Figure 3: fg006:**
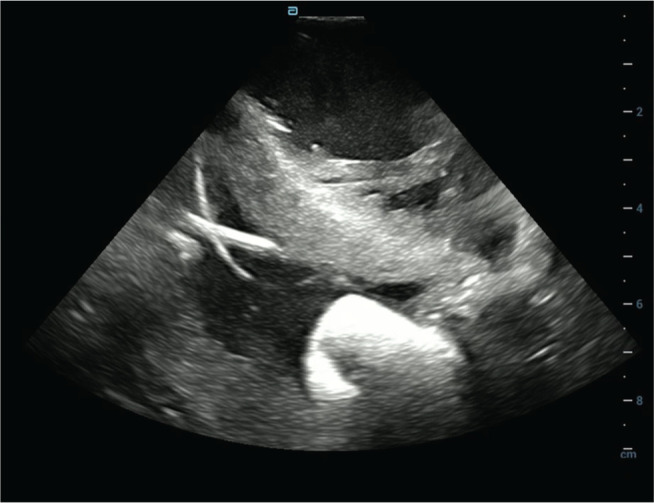
Intracardiac ultrasound showing the HD Grid mapping catheter via a transseptal approach, guiding the Sphere-9™ catheter via a retrograde aortic approach (see **Video 1**).

At baseline, the patient was in clinical VT (cycle length of 450 ms, right bundle branch block morphology, leftward axis) consistent with a mid-septal origin near the LVAD inflow cannula **(Figure 1)**. The tachycardia terminated spontaneously but was reproducibly inducible. A second non-clinical VT (cycle length of 400 ms, rightward inferior axis) was also inducible but not sustained.

High-density mapping during VT identified a slow-conduction channel along the mid-to-basal septum adjacent to the LVAD sewing ring **(Figure 4B)**, characterized by mid-diastolic potentials and late electrograms during sinus rhythm **(Figure 5, Video 2)**. Entrainment resulted in termination and change in the circuit of VT. Based on electroanatomic correlation, the target substrate encompassed the septal scar contiguous to the inflow cannula.

**Figure 4: fg007:**
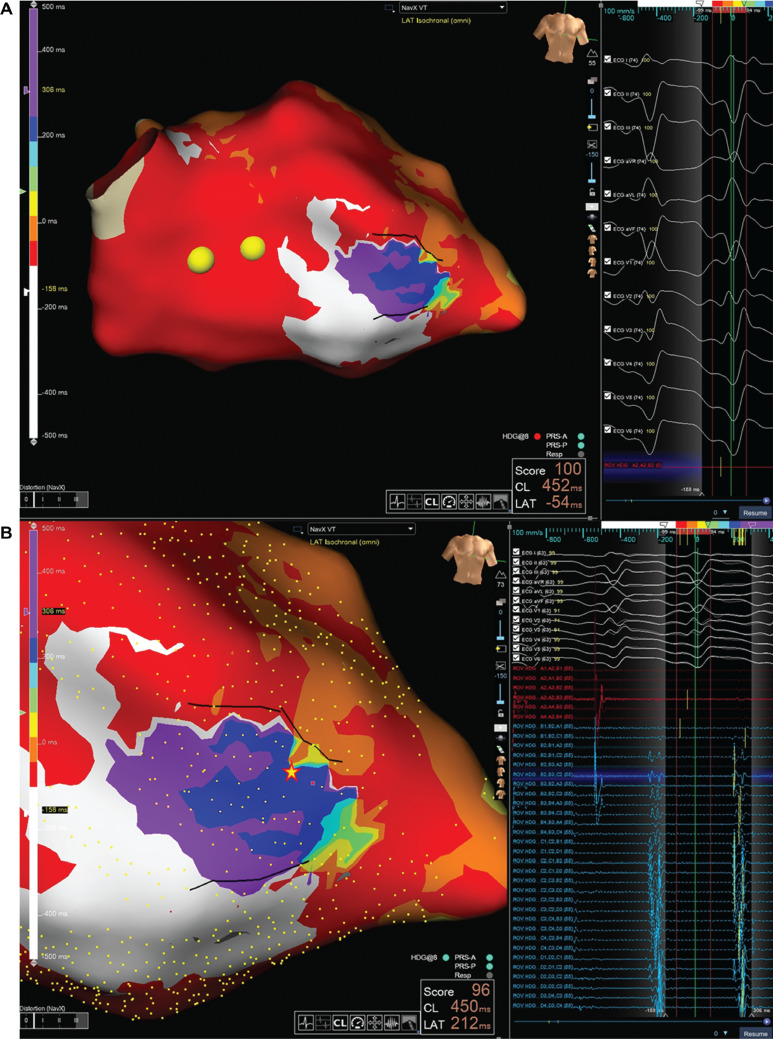
**A:** Activation mapping during ventricular tachycardia. **B:** Mid-diastolic potential during ventricular tachycardia suggestive of slow conduction channel along the mid-to-basal septum adjacent to the left ventricular assist device sewing ring (star indicates the mapping point).

**Figure 5: fg008:**
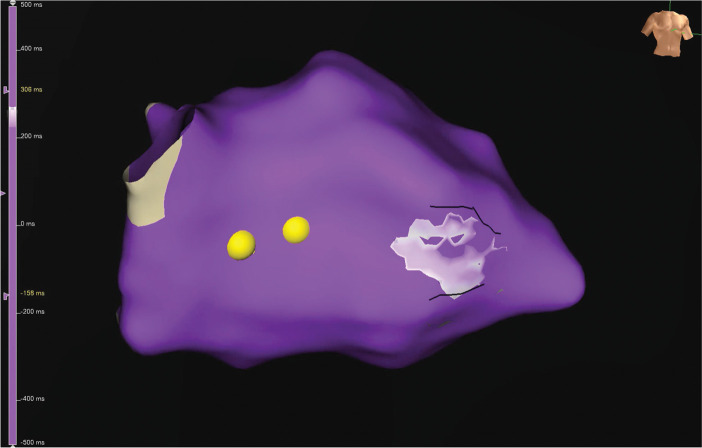
The right anterior oblique projection of a three-dimensional electroanatomic map showing signal wavefront propagation sequence during ventricular tachycardia (see **Video 2**).

Ultimately, ablation was performed using the Sphere-9™ catheter, applying an initial 30-s RF lesion, followed by four PFA applications for consolidation at each site. The clinical VT terminated abruptly during the first energy delivery. A total of 24 RF and 96 PFA applications were delivered to the targeted septal substrate extending toward the LVAD sewing ring. The hybrid RF–PFA strategy was chosen to maximize the lesion depth and durability while minimizing collateral injury risk near the conduction system and prosthetic interface.

Following lesion delivery, aggressive programmed stimulation from the LV apex and RV apex failed to induce VT. No atrioventricular block or hemodynamic compromise occurred during ablation. Post-procedure ICD interrogation showed normal device function, and programming was optimized to favor antitachycardia pacing over shocks.

The patient remained hemodynamically stable post-procedure, with stable LVAD flows and no recurrent VT on telemetry or device interrogation. CRRT was continued for renal support. Echocardiography revealed unchanged severe LV systolic dysfunction. The temporary RVAD was successfully explanted 5 days post-ablation, and the patient was extubated thereafter.

However, 7 days later, he experienced episodes of polymorphic VT of a different morphology, requiring multiple ICD shocks. He was reintubated and underwent a left stellate ganglion block, with plans for surgical sympathectomy.

***Discussion:*** This case demonstrates the first successful use of Sphere-9™ for VT ablation in an LVAD patient guided by the EnSite™ X impedance-based mapping system to overcome the EMI limitations of magnetic-based localization. EMI from the LVAD rendered Affera’s localization unreliable, but impedance-based tracking remained unaffected, allowing accurate spatial targeting. By integrating Sphere-9™ under EnSite™ guidance with ICE visualization, safe and effective ablation was achievable despite the lack of Affera visualization.

The combination of PFA and RF offers a novel approach to complex VT substrate modification, especially in regions adjacent to prosthetic structures where thermal injury risk is high. PFA’s non-thermal mechanism complements RF’s ability to produce deeper, contiguous lesions, enhancing procedural safety and efficacy.

***Conclusion:*** In patients with LVADs where EMI precludes magnetic-based mapping, Sphere-9™ ablation guided by EnSite™ impedance mapping is feasible, safe, and effective. This hybrid PFA–RF approach enables precise substrate modification in anatomically complex and device-limited settings, expanding the applicability of next-generation ablation systems to the LVAD population. Further studies are warranted to evaluate long-term lesion durability and arrhythmia-free survival in this challenging cohort.

## Supporting information

Supplementary Video 1:

Supplementary Video 2:
